# High-affinity monoclonal antibodies against the porcine epidemic diarrhea virus S1 protein

**DOI:** 10.1186/s12917-024-04091-y

**Published:** 2024-06-03

**Authors:** Qiaoli Lang, Nan Huang, Jincao Guo, Liangpeng Ge, Xi Yang

**Affiliations:** 1 Chongqing Academy of Animal Sciences, Chongqing, 402460 China; 2 National Center of Technology Innovation for Pigs, Chongqing, 402460 China; 3 Key Laboratory of Pig Industry Sciences Ministry of Agriculture, Chongqing, 402460 China; 4 School of Biological Sciences, University of Manchester, Oxford Road, Manchester, M13 9PT UK

**Keywords:** Porcine epidemic diarrhea virus (PEDV), Recombinant PEDV S1 protein, Monoclonal antibody, High affinity

## Abstract

The porcine epidemic diarrhea virus (PEDV) infection inflicted substantial economic losses upon the global pig-breeding industry. This pathogen can infect all pigs and poses a particularly high fatality risk for suckling piglets. The S1 subunit of spike protein is a crucial target protein for inducing the particularly neutralizing antibodies that can intercept the virus-host interaction and neutralize virus infectivity. In the present study, the HEK293F eukaryotic expression system was successfully utilized to express and produce recombinant S1 protein. Through quantitative analysis, five monoclonal antibodies (mAbs) specifically targeting the recombinant S1 protein of PEDV were developed and subsequently evaluated using enzyme-linked immunosorbent assay (ELISA), indirect immunofluorescence assay (IFA), and flow cytometry assay (FCA). The results indicate that all five mAbs belong to the IgG1 isotype, and their half-maximal effective concentration (EC_50_) values measured at 84.77, 7.42, 0.89, 14.64, and 7.86 pM. All these five mAbs can be utilized in ELISA, FCA, and IFA for the detection of PEDV infection. MAb 5-F9 exhibits the highest sensitivity to detect as low as 0.3125 ng/mL of recombinant PEDV-S1 protein in ELISA, while only 0.096 ng/mL of mAb 5-F9 is required to detect PEDV in FCA. The results from antigen epitope analysis indicated that mAb 8-G2 is the sole antibody capable of recognizing linear epitopes. In conclusion, this study has yielded a highly immunogenic S1 protein and five high-affinity mAbs specifically targeting the S1 protein. These findings have significant implications for early detection of PEDV infection and provide a solid foundation for further investigation into studying virus-host interactions.

**Supplementary Information:**

The online version contains supplementary material available at 10.1186/s12917-024-04091-y.

## Background

Porcine epidemic diarrhea virus (PEDV) is an enteric RNA virus that belongs to the *Coronaviridae* family [1, 2]. It is the causative agent of porcine epidemic diarrhea (PED), a diarrheal disease in swine. In the early 1970s, the swine industries of Europe and Asia experienced their first outbreak of PED, which subsequently spread to numerous other nations [3, 4]. China discovered new and highly virulent strains of PEDV in 2010, leading to its widespread dissemination across multiple countries [2, 5]. The majority of pigs infected with PEDV exhibit symptoms such as vomiting, diarrhea, and dehydration [6]. This disease can affect pigs of all ages [7, 8], but poses a particularly high morbidity and mortality risk for suckling piglets. Globally, the infection of PEDV has resulted in substantial economic losses within the pig-breeding industry [9].

PEDV is a single-stranded RNA virus with a genome size of about 28 kb, and comprises four crucial structural proteins: spike (S), envelope, membrane, and nucleocapsid proteins [4, 10–12]. Among them, the S protein plays a critical role in the process of viral infection, which plays a crucial role in facilitating virus-cell recognition events and promoting viral entry into host cells [5, 13, 14]. As with other coronaviruses, the S protein of PEDV can be split into the S1 (1–735 aa) and S2 subunits (736–1,383 aa) [15, 16]. The neutralizing epitopes are primarily located in the S1 subunit [17–19], and the S1 subunit of S protein can induce protective immunity in pigs [20, 21]. Several S-based enzyme-linked immunosorbent assays (ELISAs) have been developed [22, 23] and demonstrated to be specific for PEDV without cross-reactivity with other swine coronaviruses. As a result, an S-based ELISA is more suitable for developing a specific detection method for PEDV.

Although several monoclonal antibodies have been developed against the PEDV-S protein [17, 24–28], the researchers primarily focus on characterizing mAbs based on their specificity, neutralization capacity, or therapeutic potential, the quantitative analysis of the affinity activities of these monoclonal antibodies (mAbs) has been scarcely conducted. Current detection methods for PEDV suffer from issues such as low sensitivity, specificity, and difficulties in early detection. However, high-affinity antibodies can enhance the sensitivity of antibody-based viral detection by effectively binding to viral surface antigens, generating strong signals even at low viral concentrations. Thus, high-affinity antibodies are crucial for the sensitive detection of target proteins, which is particularly important in early PEDV infection diagnosis. In this study, we utilized a eukaryotic expression system to produce PEDV-S1 recombinant protein for immunization of mice in order to generate high-affinity antibodies against PEDV. Through quantitative analysis, five mAbs with high affinity were generated and their half-maximal effective concentration (EC_50_) values reached the ≈ 10^–13^ M scale. These mAbs have the potential to be utilized for the early identification of PEDV infection, and our research provides a solid foundation for further investigation.

## Results

### The expression and purification of PEDV-S1 protein

**Fig. 1:**
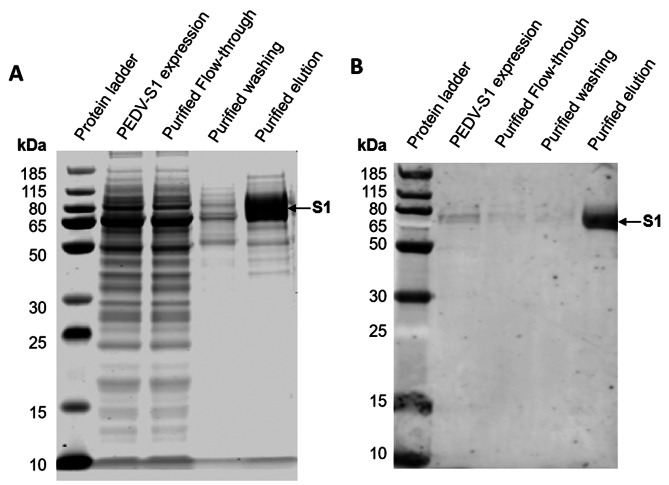
Expression and purification of recombinant PEDV-S1 protein. **A** The recombinant PEDV-S1 protein was expressed in HEK293F cells and subsequently purified using Ni^2+^ column affinity chromatography. The protein samples obtained during the purification process were subjected to analysis using SDS-PAGE and scanned using the Odyssey CLX platform. **B** The expression and purification of the recombinant PEDV-S1 protein were confirmed through western blotting, employing an anti-His antibody for detection

The gene encoding S1 subunit (234–736 amino acids) of PEDV S protein was cloned into plasmid pcDNA3.4 with a six-histidine (6×His)-tag at its C-terminus. The recombinant vector pcDNA3.4-S1 was transfected into HEK239F cells to express the target protein, which was then purified using the Ni^2+^ column affinity chromatography to obtain highly pure PEDV-S1 protein (approximately 65 kDa) (Fig. 1A). Western blot analysis of PEDV-S1 was subsequently performed using anti-His antibody (Fig. 1B).

### Preparation and characterization of PEDV-S1 monoclonal antibodies

**Fig. 2:**
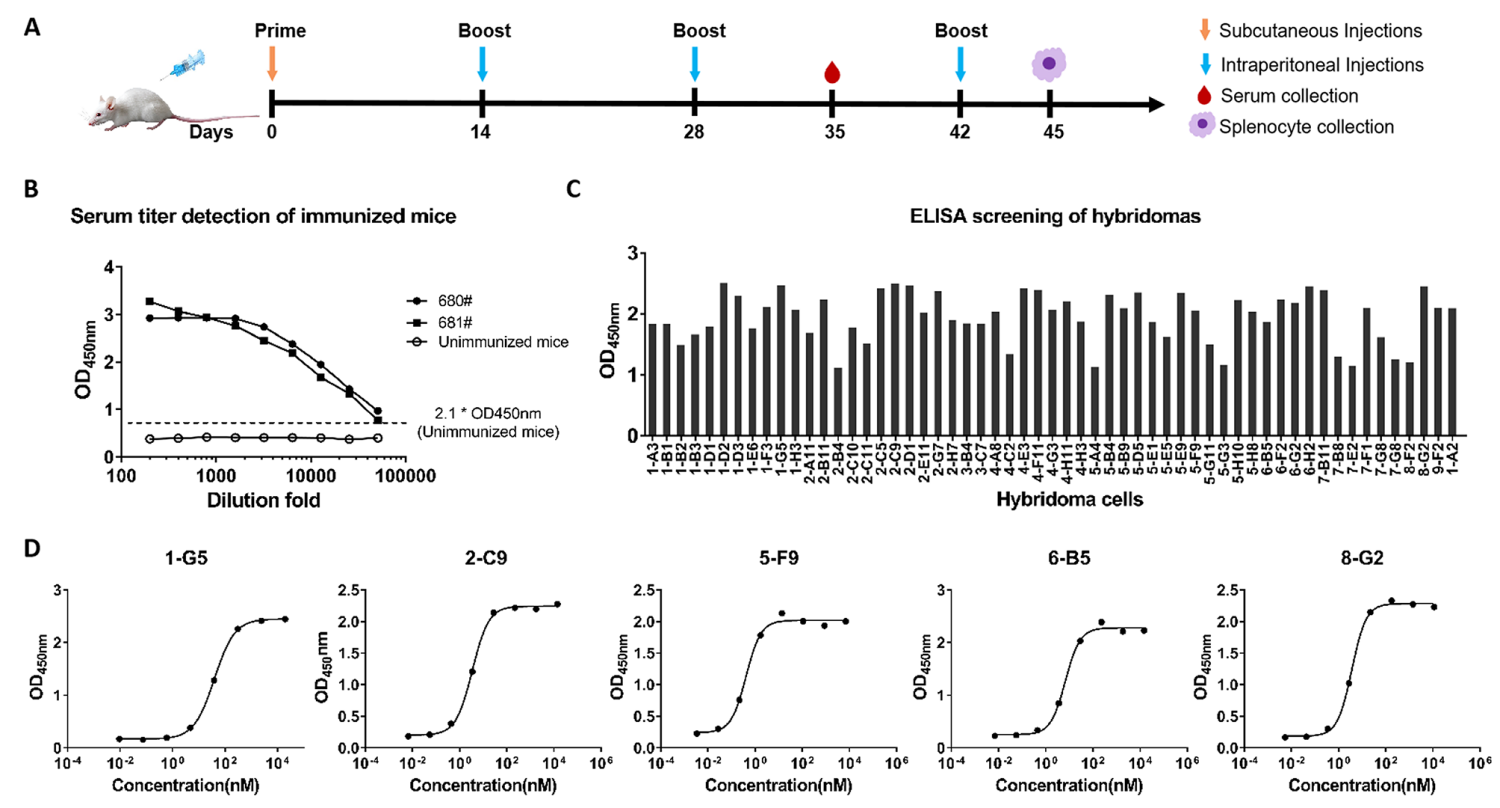
Preparation of PEDV-S1 mAbs. **A** Immunization schedule for mice. **B** Immunization of mice with the PEDV-S1 protein. **C** ELISA screening of hybridomas for PEDV-S1 protein mAbs. **D** The EC_50_ of the prepared mAbs against PEDV-S1 protein was determined using an indirect ELISA, with the hybridoma supernatant serving as the primary antibody. The mAb concentrations in the hybridoma supernatant were determined by an ELISA kit (Thermo Scientific, USA)

Two female BALB/c mice were immunized with recombinant PEDV-S1 protein at a 2-week interval. After the third immunization, the antibody titers of the two immunized mice reached 1:10,000 (Fig. 2B). Following fusion of splenocytes from immunized mice with SP2/0 cells, hybridoma clones were screened and cultivated in HAT semi-solid medium. Positive hybridoma clones were identified using cell culture media and an indirect ELISA, resulting in successful acquisition of over 47 positive hybridoma clones (OD_450nm_ > 1.0) as depicted in Fig. 2C. To obtain stable hybridoma clones, we performed five passages and generated 11 stable antibody-secreting hybridoma clones (Supplementary Fig. 1). These clones were screened for their reactivity against PEDV-S1 using indirect ELISA, and the top five clones, namely 1-G5, 2-C9, 5-F9, 6-B5, and 8-G2, were selected based on their highest titers. The five hybridoma cell lines demonstrating the highest reactivity towards PEDV-S1 were subjected to further testing, resulting in the determination of their EC_50_ values. These values were as follows: 1-G5 (12.71 ng/mL or 84.77 pM), 2-C9 (1.114 ng/mL or 7.42 pM), 5-F9 (0.1332 ng/mL or 0.89 pM), 6-B5 (2.196 ng/mL or 14.64 pM), and 8-G2 (1.18 ng/mL or 7.86 pM), as shown in Fig. 2D.

### Characterization of mAbs against PEDV-S1

**Fig. 3:**
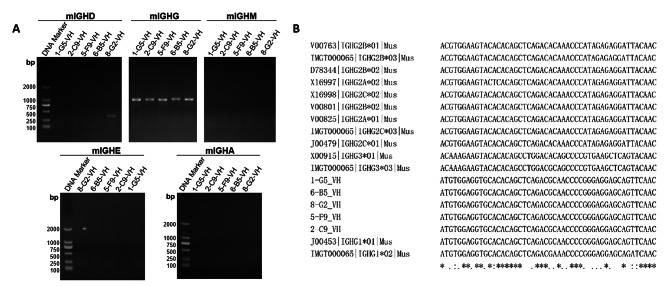
Identification of PEDV-S1 mAb subtypes. In this study, five hybridoma cell lines capable of stable secretion of PEDV-S1 antibodies were generated. **A** The heavy chain variable region and CH2 region of the five monoclonal antibodies (mAbs) were amplified using PCR. The subtypes of these antibodies were determined through PCR analysis. **B** PCR products were subjected to sequence alignment to compare the nucleotide or amino acid sequences

**Fig. 4:**
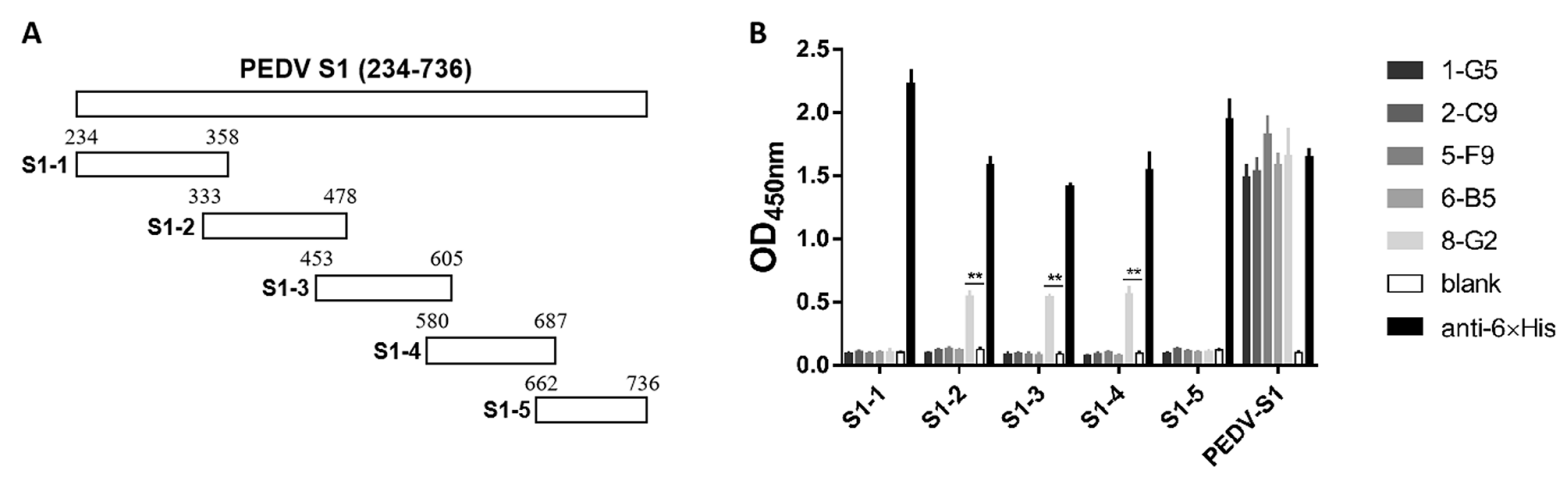
Epitope analysis of PEDV-S1 mAbs. **A** The full-length PEDV-S1 protein was cut into five segments: S1-1, S1-2, S1-3, S1-4, and S1-5, each with a 25-amino acid overlap. **B** Identify the epitopes of the five mAbs by ELISA. Data were analyzed using a Student’s t-test with two-tailed independents, **p < 0.05

The antibody type was identified by PCR amplification of the heavy chain variable and CH2 regions, revealing that all five mAbs were mouse IGHG isotypes (Fig. 3A), with further sequencing confirming them as mouse IGHG1*01 subtypes (Fig. 3B). The epitopes of the five mAbs were identified by cleaving the full-length PEDV-S1 protein into five segments, namely S1-1, S1-2, S1-3, S1-4, and S1-5 (Fig. 4A), with a 25 amino acid overlap between adjacent sections. These segments were subsequently incorporated into plasmid pcDNA3.4 and expressed in the HEK239F eukaryotic expression system. The resulting truncated proteins were utilized as coating antigens in an indirect ELISA to determine the epitopes of the five mAbs. The findings indicated that 8-G2 exhibited significant binding affinity towards the truncated proteins S1-2, S1-3, and S1-4, while the remaining four mAbs did not exhibit any binding affinity towards these truncated proteins. Consistently, mAb 5-F9 demonstrated the highest binding activity in its interaction with the full-length PEDV-S1 protein (Fig. 4B).

### Applications of the mAbs for the detection of PEDV infection

**Fig. 5:**
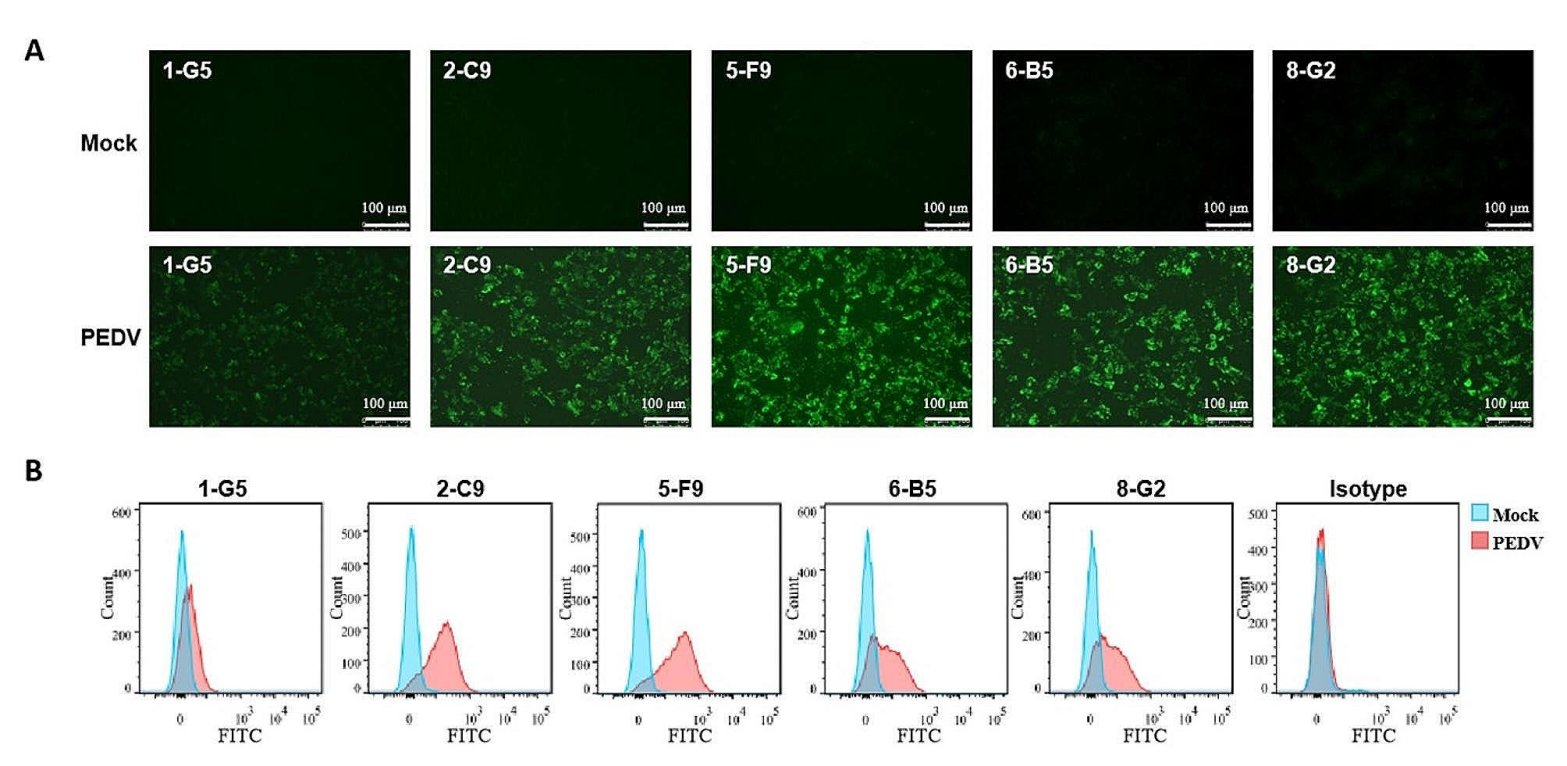
Functional analysis of the mAbs by IFA and FCA. The Vero E6 cells were infected with PEDV at MOI = 0.1 and collected 48 h post-infection. **A** Both PEDV-infected and mock-infected Vero E6 cells were utilized and subjected to incubation with each of the five prepared mAbs, respectively. Subsequently, the cells were fixed, and fluorescent microscopy was used to visualize them after incubation with FITC-labeled secondary antibodies. B The Vero E6 cells, whether infected with PEDV or not, were individually stained with the five mAbs and the mouse IgG1 hybridoma supernatant, subsequently incubated with a FITC-labeled goat anti-mouse IgG secondary antibody (Abcam, UK). The cells were then resuspended in 2% FBS-PBS, and the BD FCAVerse™ flow cytometer was utilized to conduct the analysis

This study assessed the suitability of the five mAbs for detecting PEDV infection through immunofluorescence assay (IFA) and flow cytometry assay (FCA). Vero E6 cells, either infected with PEDV or uninfected, were fixed and subjected to IFA with these five mAbs, respectively. The results showed that all mAbs specifically bound to PEDV-infected Vero E6 cells while showing no binding affinity towards normal Vero E6 cells (Fig. 5A). Furthermore, FCA analysis revealed that all tested mAbs effectively recognized the presence of PEDV virus, with 5-F9 exhibiting the highest binding activity among them (Fig. 5B). The characteristics of these antibodies, such as their EC_50_ values, subtype, epitope, and performance in IFA and FCA, were displayed in Table 1. These results indicate that the five mAbs have potential utility in ELISA, FCA, and IFA for the detection of PEDV infection.

**Table 1:** Characterization and applications of the mAbs against PEDV-S1

mAbs	Subtypes	EC_50_ (pM)	Types of epitope	IFA (Mean gray value)^1^	FCA (FITC-A Mean)^2^
1-G5	IGHG1*01	84.77	conformational epitopes	6.138	28
2-C9	IGHG1*01	7.42	conformational epitopes	45.221	221
5-F9	IGHG1*01	0.89	conformational epitopes	76.016	393
6-B5	IGHG1*01	14.64	conformational epitopes	52.396	110
8-G2	IGHG1*01	7.86	linear epitopes	61.029	101

1: The gray value of IFA was analyzed by ImageJ software (v1.8.0); 2: Mean FITC-A mean: Mean fluorescence intensities; Mock-cell values were subtracted from the values in all assays.

### Sequencing, expression, and characterization of the mAb 5-F9

**Fig. 6:**
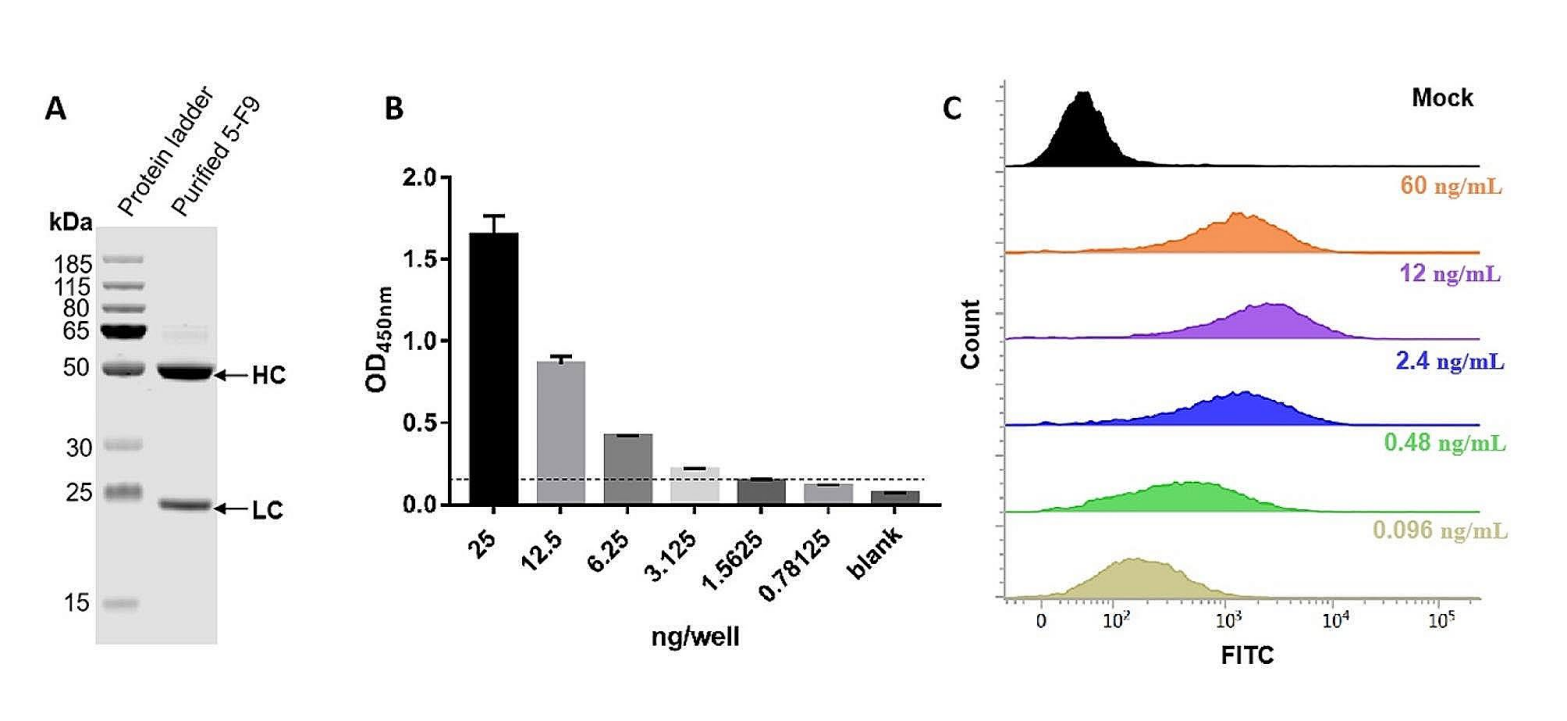
Characterization of the purified mAb 5-F9. **A** SDS-PAGE analysis of purified mAb 5-F9. **B** Sensitivity of mAb 5-F9 for detection of recombinant PEDV-S1 protein with ELISA. **C** The sensitivity of mAb 5-F9 for the detection of PEDV virus with FCA

The mAb 5-F9, which exhibited the highest binding activity to both recombinant PEDV-S1 protein and PEDV virus, was selected for further sequencing, expression, and characterization. Using the IGBLAST database (https://www.ncbi.nlm.nih.gov/igblast/), we analyzed the localization of the variable regions CDR1, CDR2, and CDR3 in mAb 5-F9 as listed in Table 2. The heavy chain and light chain gene sequences of mAb 5-F9 were inserted into plasmid pcDNA3.4 and expressed in the HEK239F eukaryotic expression system. Then, recombinant antibody 5-F9 was isolated using protein A chromatography method. High-purity recombinant antibody 5-F9 was successfully obtained in this study (Fig. 6A). The sensitivity of mAb 5-F9 in detecting recombinant PEDV-S1 protein was assessed by indirect ELISA. Different amounts of recombinant PEDV-S1 protein (25, 12.5, 6.25, 3.125, 1.5625, and 0.78125 ng) were coated onto each well of the plate. The findings indicate that mAb 5-F9 can detect recombinant PEDV-S1 protein at concentrations as low as 3.125 ng/well (0.3125 ng/mL) when used at a concentration of 50 ng/well (Fig. 6B). Furthermore, the sensitivity of mAb 5-F9 was also evaluated for detecting PEDV virus, utilizing FCA analysis to titrate pure mAb 5-F9 (at concentrations of 60, 12, 2.4, 0.48, and 0.096 ng/mL) on PEDV-infected Vero E6 cells. The results demonstrate that mAb 5-F9 has the capability to detect concentrations as low as 0.096 ng/mL (Fig. 6C).

**Table 2:** Analysis of the 5-F9 mAb variable region

	Locus	CDR1	CDR2	CDR3
5-F9-VH	IGHV1-19*01	GYTFTDYY	IDPYNGGT	ARVGYNGYWYFDV
5-F9-Vκ	IGKV16-104*01	KSISKY	SGS	QQHNESPIT

## Discussion

PEDV is a highly contagious gastrointestinal disease that causes acute diarrhea in swine, with potentially fatal consequences for young piglets. The global pig industry has suffered significant economic losses due to PEDV infection, highlighting the critical importance of early detection. The S protein of PEDV is a surface protein located on the viral envelope, which plays a crucial role in mediating attachment and entry into the host’s target cells. It possesses several epitopes and highly antigenic index regions that elicit the production of neutralizing antibodies [29–31]. Notably, numerous B cell epitopes responsible for inducing the neutralizing antibody responses are predominantly situated within the S1 subunit [17–19], making it an ideal target for developing novel genetically modified vaccines and therapeutic antibodies [20, 32–36].

Due to the significant impact of PEDV on the pig industry, early detection is crucial. However, the identification of the virus at an early stage presents substantial challenges due to its limited presence. Therefore, it is imperative to employ pathogen detection techniques with high sensitivity. Achieving a highly sensitive ELISA always requires high-affinity antibodies. Unfortunately, there has been a lack of comprehensive assessment regarding antibody affinity in PEDV detection [17, 24–28], which may potentially impact the limited sensitivity of ELISA.

In this study, to generate high-affinity antibodies against PEDV, we immunized mice with PEDV-S1 recombinant protein expressed by using a eukaryotic expression system (Fig. 1). Through hybridoma fusion and quantitative analysis, five mAbs with high affinity were identified (Fig. 2). Our results showed that these five mAbs exhibited high affinity for the PEDV-S1 recombinant protein, with EC_50_ values ranging from 0.89 to 84.77 pM. Notably, mAb 5-F9 demonstrated the strongest binding activity. The suitability of these five mAbs for detecting PEDV infection was assessed through IFA and FCA analyses (Fig. 5). However, the results of IFA and FCA analysis showed inconsistency for 6-B5 and 8-G2, suggesting that fixation of the virus during IFA, which was not done in FCA, may have affected the binding characteristics of these antibodies. Specifically, 8-G2 and 6-B5 demonstrated higher binding activity to fixed virus compared to live virus, resulting in the discrepancy between IFA and FCA results. Additionally, 2-C9 and 8-G2 showed similar EC_50_ values but different FCA values. This may be attributed to the structural differences between the S1 subunit used for antibody selection and the natural PEDV virus. Moreover, 8-G2 binds to a different conformational epitope compared to the other four antibodies (Fig. 4), potentially leading to weaker binding to PEDV S protein in its natural conformation.

In comparison to previously reported antibodies for PEDV detection and treatment, the antibodies obtained in this study demonstrate two significant advantages. Firstly, we performed affinity testing and determined the binding activity of the antibodies to PEDV, revealing higher affinity compared to previously reported PEDV antibodies. For instance, Bao et al. [25] produced an antibody that exhibited binding to PEDV S protein at a concentration of 1 µg/mL, while Yang et al. [37] developed an antibody capable of detecting PEDV-N protein as low as 0.487 ng/mL. In contrast, mAb 5-F9, generated through the HEK293F eukaryotic expression system, exhibited the ability to detect PEDV-S1 protein at concentrations as low as 0.3125 ng/mL in ELISA. Furthermore, at a concentration of just 0.096 ng/mL, mAb 5-F9 successfully identified PEDV-infected cells in FCA, highlighting its exceptional sensitivity. Secondly, we constructed a eukaryotic expression vector and successfully expressed and purified the antibody using HEK293F cells (Fig. 6). The obtained antibody retained high affinity, indicating reproducibility and stability, making the mAb suitable for commercial production and widespread application. However, this study still has some shortcomings that require further research. In the course of screening for high-affinity antibodies against PEDV, although eukaryotic expression systems provided proteins more natural than those from prokaryotic systems [38, 39], there was also some discrepancy observed between the ELISA results obtained with the PEDV-S1 protein and the IFA results obtained with the PEDV virus. The findings suggest that the PEDV-S1 protein exhibits a noticeable divergence from the genuine viral architecture. Therefore, the acquisition of an antigen that closely mimics the virus’s natural structure and possesses strong immunogenic properties remains a challenge. Furthermore, the application of antibodies to pig samples infected with PEDV has not been tested in this study. The validation of antibody effectiveness in early PEDV diagnosis will continue to be a central focus of our future research endeavors.

## Conclusion

In this study, highly immunogenic PEDV-S1 protein was successfully generated by using the HEK293F eukaryotic expression system. Five high-affinity mAbs with robust binding affinity towards the PEDV-S1 protein were successfully acquired, among which mAb 5-F9 exhibited exceptional purity, affinity, and sensitivity. Our research provides a solid foundation for investigating virus-host interactions and early detection of PEDV infection.

## Methods

### Mice, cell lines, virus, and plasmids

The Institutional Animal Care and Use Committee at the Chongqing Academy of Animal Sciences approved the animal experiments (Licence No. SYXK (Yu) 2022-0010). Six-week-old female BALB/c mice for the experiment were supplied by Beijing Vital River Laboratory Animal Technology Co., Ltd. All animal experiments conform to the relevant provisions of animal welfare. By using CO_2_ euthanasia, mice were sacrificed with a minimum of suffering after completion of the study. The Vero-E6 cell line (ATCC CRL-1586) is derived from African green monkey kidney tissue and exhibits epithelial morphology. The Dulbecco’s Modified Eagle Medium (Procell Life Science & Technology Co., Ltd., China) was used to culture the Vero E6 cell, along with 10% fetal bovine serum (FBS, Biological Industries, Israel) and 1% penicillin-streptomycin solution. Vero E6 cells were used to multiply and titrate the PEDV-SP-C strain [40]. The pcDNA3.4 plasmids served as the expression vectors of HEK239F eukaryotic expression system.

### Recombinant PEDV-S1 protein expression and purification

The gene encoding the PEDV S1 subunit (234–736 amino acids) and its corresponding segments S1-1 (234–358 amino acids), S1-2 (333–478 amino acids), S1-3 (453–605 amino acids), S1-4 (580–687 amino acids), and S1-5 (662–736 amino acids), derived from the spike protein of the PEDV AJ1102 strain (GenBank: AFQ37598.1), were synthesized and inserted into the pcDNA3.4 eukaryotic expression vector (Invitrogen, USA) by Suzhou GENEWIZ Biotechnology. The full-length PEDV S1 subunit was tagged with a His tag at the N-terminus. Segments S1-1 to S1-5 were tagged with a His tag at the N-terminus and a mouse IgG2a Fc tag at the C-terminus, with sequencing confirming the accuracy of the recombinant plasmids. These plasmids were transfected into HEK239F cells (Thermo Fisher Scientific, USA) for protein expression. Ni^2+^ column affinity chromatography was employed to purify the recombinant protein (Cytiva, USA) according to the guidelines. SDS-PAGE analysis confirmed the purity of the purified recombinant protein. Western blot analysis was conducted with an anti-His primary antibody to confirm the presence of the target protein.

### Immunization of mice

The immunization procedure for mice included the subcutaneous injection of 200 µg of PEDV-S1 protein with Freund’s complete adjuvant (Sigma-Aldrich, USA) for the primary immunization in six-week-old female BALB/c mice, followed by two intraperitoneal booster immunizations with 200 µg of PEDV-S1 protein using Freund’s incomplete adjuvant (Sigma-Aldrich, USA) at a two-week interval.

### Selection of high-affinity mAbs

One week post the third immunization, mouse serum was collected for the measurement of antibody titers via indirect ELISA using the purified PEDV-S1 protein. Three days prior to the hybridoma fusion, mice with a titer of 1:10,000 following triple immunization were intraperitoneally injected with 200 µg of purified PEDV-S1 protein without adjuvant. Splenocytes from these immunized mice were isolated and fused with SP2/0 cells at a 5:1 ratio, utilizing the bipolar pulsed electric field electrofusion technique [41]. The hybridoma clones were screened and cultured with HAT semi-solid medium. The cell culture medium was subsequently assessed using indirect ELISA coating with PEDV-S1 protein to detect positive hybridoma clones. Clones with an OD_450nm_ exceeding 1.0 were further cultured through five stable passages to establish hybridoma cells that consistently produced the desired antibody. Antibody titers in the supernatants from these hybridomas were then quantified via indirect ELISA on PEDV-S1 coated plates, employing serial dilutions ranging from 4- to 65,536-fold to isolate high-titer clones. The concentrations of mAbs in these selected clones were quantified by an ELISA kit (Thermo Scientific, USA), and the EC_50_ of these mAbs were calculated utilizing GraphPad Prism 7.0 software.

### Identification of mAb subtypes

The identification of the mAb antibody type was accomplished through PCR amplification of the variable region and CH2 region of the mAb heavy chains, using the method and primers described by Huang et al. [42]. After sequencing, BLAST tool (https://blast.ncbi.nlm.nih.gov/) was utilized to identify the mAb subtypes.

### Epitope mapping by indirect enzyme-linked immunosorbent assay (ELISA)

The ELISA plate wells were coated with 50 ng of purified S1-1, S1-2, S1-3, S1-4, S1-5, and the full-length PEDV-S1 protein in coating buffer, followed by overnight incubation at 4 °C. The wells were then blocked with 2% BSA at 37 °C for 2 h. After three washes with PBST, the hybridoma supernatant of the 5 mAbs and 1% BSA as a blocking agent were added and incubated for additional 2 h at 37 °C. Subsequently, an anti-mouse IgG (Fab)-peroxidase-conjugated goat antibody (Sigma-Aldrich, USA) at a 1:5,000 dilution was added for antigen epitope analysis, and an anti-His primary antibody (Abcam, UK) at the same dilution was used to assess the antigen’s activity. The reaction was terminated with the addition of 3,3′,5,5′-tetramethylbenzidine (TMB, Solarbio) and 2 M H_2_SO_4_. Absorbance was measured at OD_450nm_ using a microplate reader (Epoch, BioTek Instruments, Inc.). The results were reported as a sample-to-negative (S/N) ratio, calculated as the ratio of OD values of the sample to the OD value of the negative control. A S/N ratio equal to or greater than 2.1 was considered indicative of a positive sample.

### Sequencing, expression, and purification of the mAbs

The sequencing of hybridoma immunoglobulin genes were conducted by Nanjing Detai Bioengineering Co., Ltd. The localization of the variable regions, CDR1, CDR2, and CDR3 regions, was analyzed using the IGBLAST database (https://www.ncbi.nlm.nih.gov/). The mAb heavy chain gene was optimally synthesized and cloned into a plasmid called pcDNA3.4-VH, while the mAb light chain gene was optimally synthesized and cloned into another plasmid called pcDNA3.4-Vκ by Suzhou GENEWIZ Biotechnology. The correctness of the resulting plasmids, pcDNA3.4-VH and pcDNA3.4-Vκ, was confirmed by sequencing. These plasmids were then co-transferred into the HEK239F eukaryotic expression system at a ratio of 1:1. Then, the cell supernatant was purified via protein A chromatography (Cytiva, USA).

### Enzyme-linked immunosorbent assay (ELISA)

The ELISA plate wells were coated with 25 ng of PEDV-S1 protein in coating buffer overnight at 4 °C, and subsequently blocked with 2% BSA at 37 °C for 2 h to determine the antibody titer of immunized mice or positive hybridoma cells. After three washes with PBST, the diluted serum or hybridoma supernatant was added and incubated for 2 h at 37 °C. Subsequently, anti-mouse IgG (H + L)-peroxidase goat antibody (Sigma-Aldrich, USA) to a ratio of 1:5,000 was added and incubated at 37 °C for 1 h. The reaction was then stopped by the addition of 3,3′,5,5′-Tetramethylbenzidine (TMB, Solarbio) and 2 M H_2_SO_4_. A microplate reader was used to measure the absorbance at OD_450nm_ (Epoch, BioTek Instruments, Inc.). The results were reported as a sample-to-negative (S/N) ratio, calculated as the ratio of OD values of the sample to the OD value of the negative control. A S/N ratio equal to or greater than 2.1 was considered indicative of a positive sample.

### Indirect immunofluorescence assay (IFA)

PEDV was introduced into Vero E6 cells at a multiplicity of infection (MOI) of 0.1, and the cells were harvested 48 h post-infection. Following infection with PEDV or a sham infection, Vero E6 cells were fixed with 4% paraformaldehyde at room temperature for 30 min and subsequently blocked with 5% BSA at 37 °C for 1 h. The cells were then incubated with hybridoma supernatant at 4 °C overnight. After being washed thrice with PBS, Goat anti-mouse IgG secondary antibody (FITC-labeled) from Abcam (UK) to a ratio of 1:1,000 was added and incubated at 37 °C for 1 h. Finally, the cells were visualized under fluorescence microscopy (DMi8, Leica Microsystems).

### Analysis of flow cytometry

Vero E6 cells were infected with PEDV at a MOI of 0.1 and harvested 48 h post-infection. The Vero E6 cells, obtained from either PEDV-infected or mock-infected cultures, were subsequently subjected to mAb staining at 4 °C for 1 h. After three washes with 2% FBS-PBS, a FITC-labeled goat anti-mouse IgG secondary antibody from Abcam (UK) to a ratio of 1:500 was added and incubated at 4 °C for 1 h. The cells were then suspended in 2% FBS-PBS and analyzed using the BD FCAVerse™ flow cytometer.

### Statistical analysis

Statistical analysis was performed using the GraphPad Prism 7.0 software. All of the data were analyzed using a Student’s t-test with two-tailed independents. Significant *P*-values were considered at *p* < 0.05.

### Electronic supplementary material

Below is the link to the electronic supplementary material.

Supplementary Material 1

Supplementary Material 2

Supplementary Material 3

## Data Availability

The gene sequences of variable regions of mAb 5-F9 generated and/or analysed during the current study are available in the NCBI repository, GenBank: PP764240 and PP764241.

## Abbreviations

PEDV: Porcine epidemic diarrhea virus
PED: Porcine epidemic diarrhea
mAbs: Monoclonal antibodies
FCA: Flow cytometry assay
IFA: Indirect immunofluorescence assay
EC_50_: Measured half of the effective concentration
qPCR: Quantitative real-time PCR
FBS: Fetal bovine serum
PBS: Phosphate buffered saline
MOI: Multiplicity of infection
