# Supplementary material for: High-affinity monoclonal antibodies against the porcine epidemic diarrhea virus S1 protein
Source: BMC Vet Res. 2024 Jun 3;20:239. doi: 10.1186/s12917-024-04091-y (PMC11145877; doi:10.1186/s12917-024-04091-y)
Supplement: Supplementary file 1 — Supplementary Material 1 [file 12917_2024_4091_MOESM1_ESM.docx]

High-affinity monoclonal antibodies against the porcine epidemic diarrhea virus S1 protein

Qiaoli Lang^^[[1]](#footnote-0)^^^,^^^[[2]](#footnote-1)^^^,^^^[[3]](#footnote-2)^^, Nan Huang^1,2,3^, Liangpeng Ge^1,2,3^^[[4]](#footnote-3)^* and Xi Yang^1,2,3*^

**The original images of Fig. 1A**

**
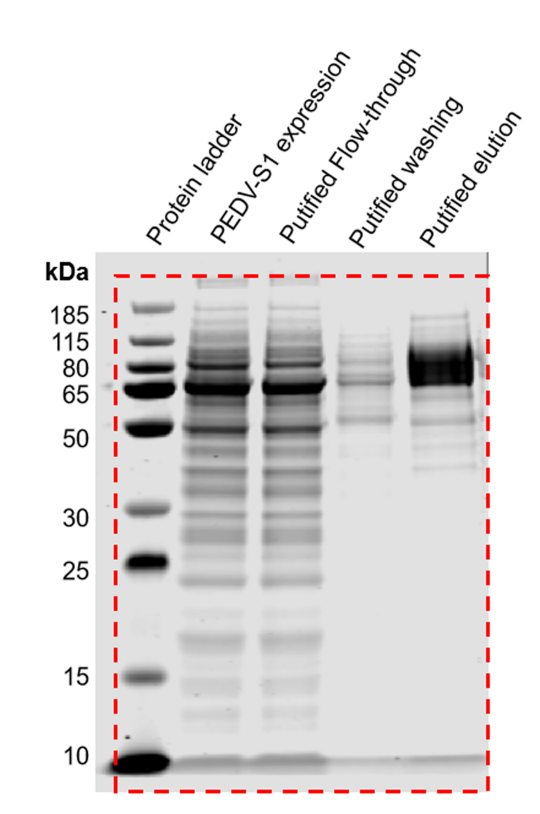
**

**The original images of Fig. 1B**

**
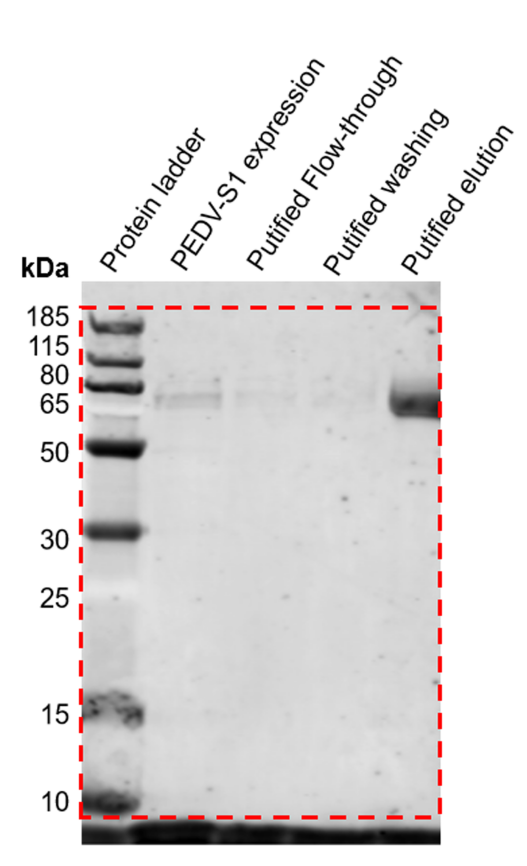
**

**The original images of Fig. 3A**

**
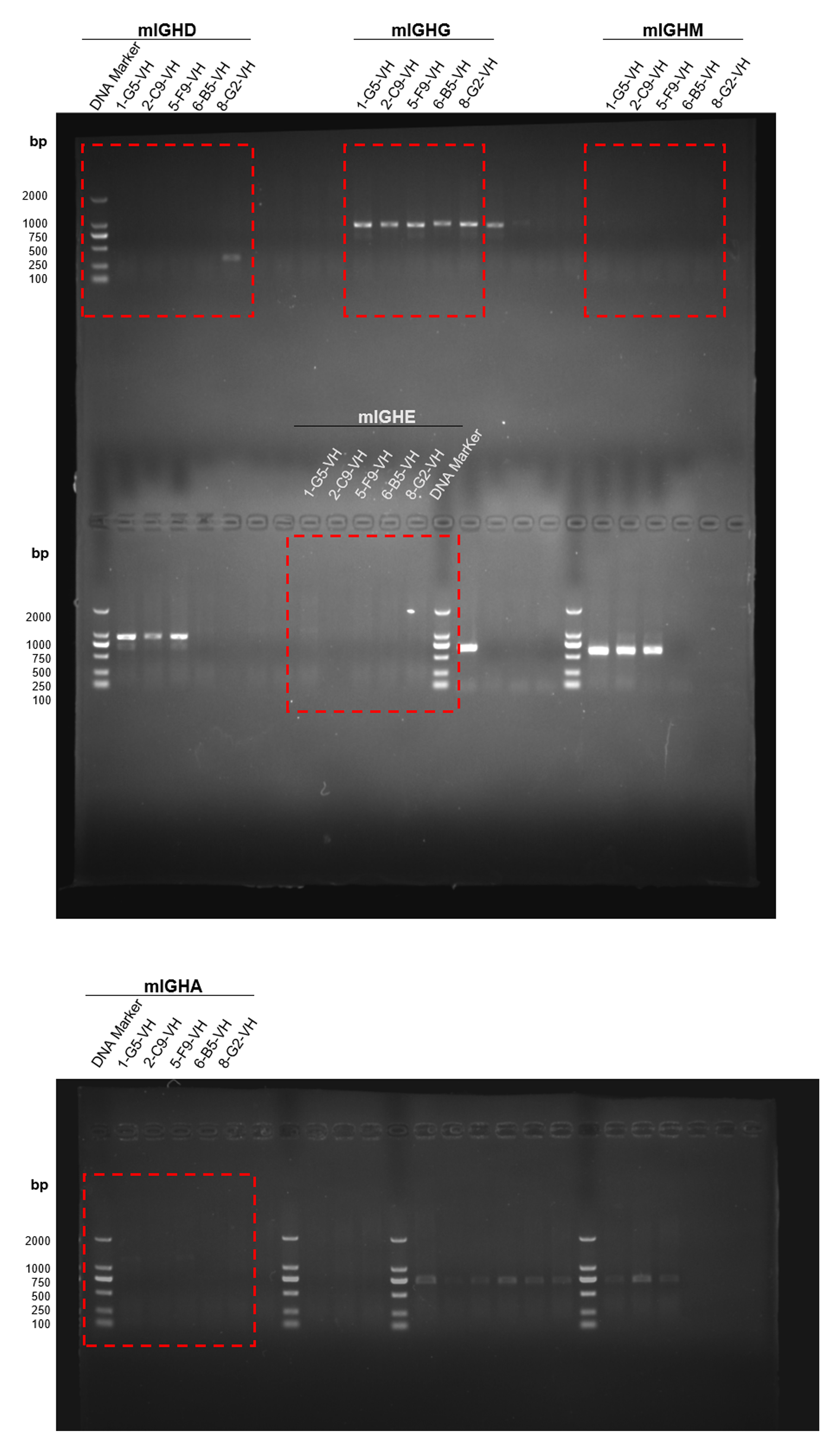
**

**The original images of Fig. 6A**

**
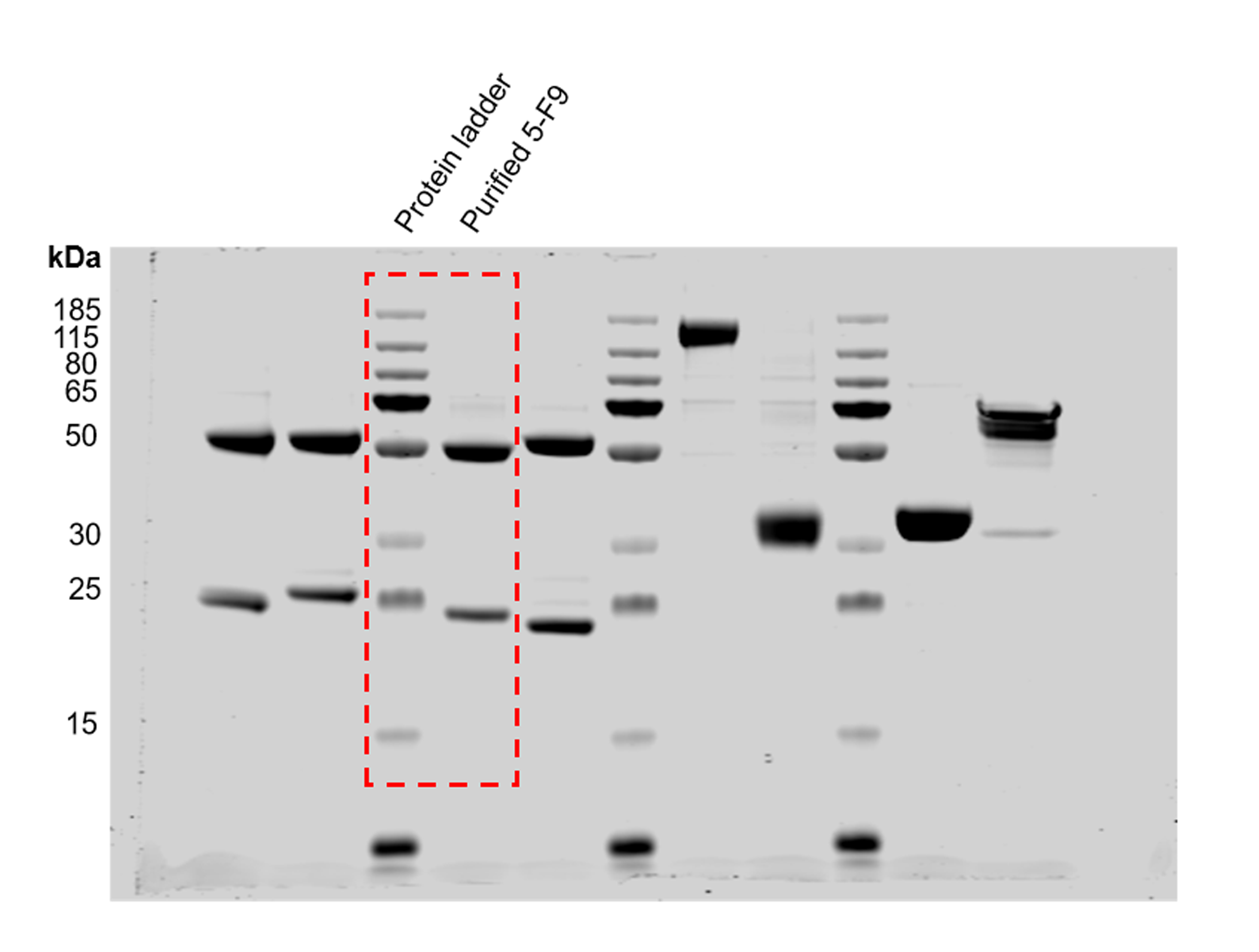
**

1. * Correspondence:

   Liangpeng Ge

   geliangpeng1982@163.com

   Xi Yang

   406162197@qq.com

   Chongqing Academy of Animal Sciences, Chongqing, 402460, China [↑](#footnote-ref-0)
2. National Center of Technology Innovation for Pigs, Chongqing, 402460, China [↑](#footnote-ref-1)
3. Key Laboratory of Pig Industry Sciences Ministry of Agriculture, Chongqing 402460, China [↑](#footnote-ref-2)
4. [↑](#footnote-ref-3)
