# Supplementary figures and images for: High-affinity monoclonal antibodies against the porcine epidemic diarrhea virus S1 protein
Source: BMC Vet Res. 2024 Jun 3;20:239. doi: 10.1186/s12917-024-04091-y (PMC11145877; doi:10.1186/s12917-024-04091-y)

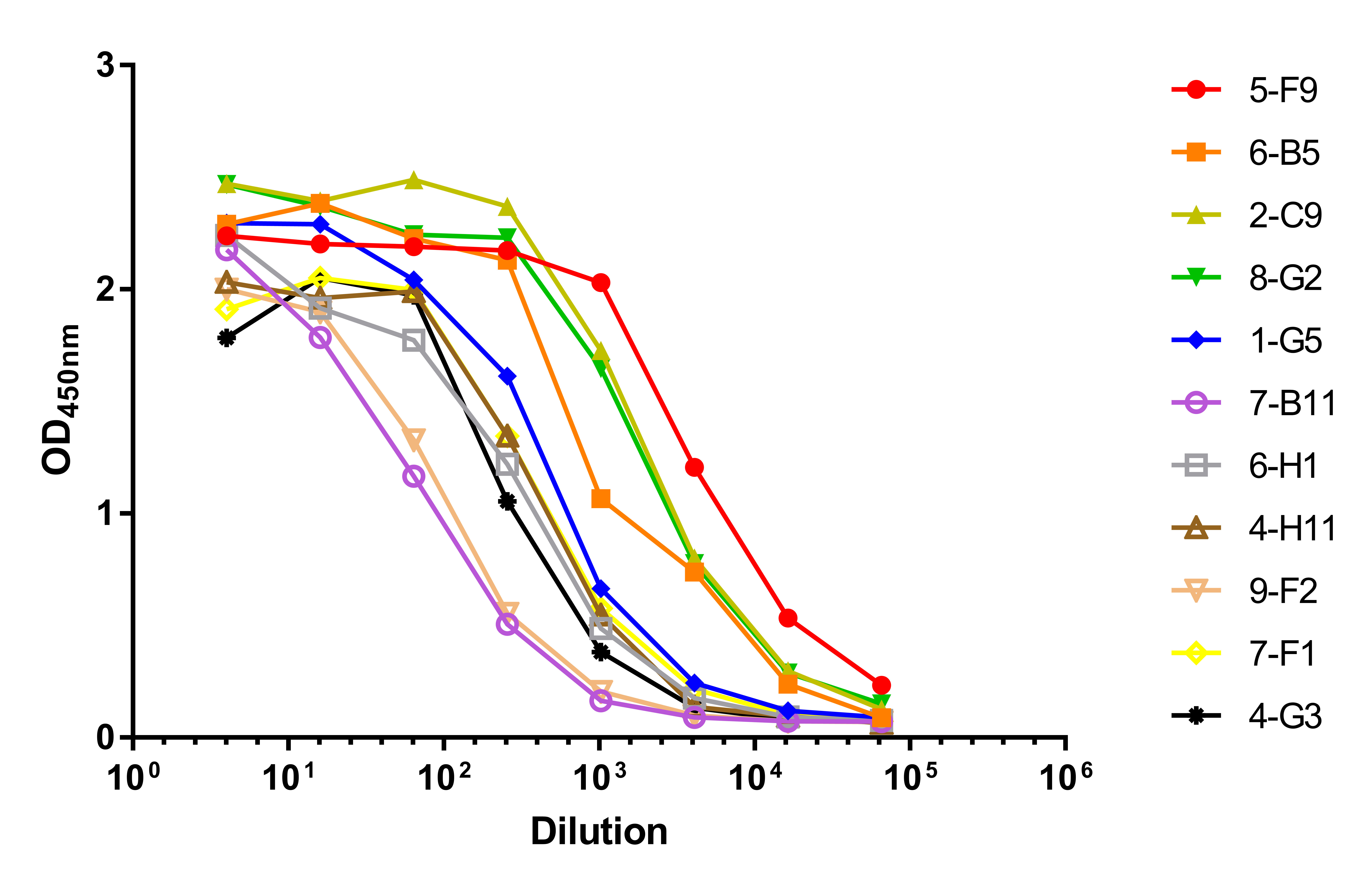

Supplement: Supplementary file 2 — Supplementary Material 2 [file 12917_2024_4091_MOESM2_ESM.tif]
