# Supplementary material for: High-affinity monoclonal antibodies against the porcine epidemic diarrhea virus S1 protein
Source: BMC Vet Res. 2024 Jun 3;20:239. doi: 10.1186/s12917-024-04091-y (PMC11145877; doi:10.1186/s12917-024-04091-y)
Supplement: Supplementary file 3 — Supplementary Material 3 [file 12917_2024_4091_MOESM3_ESM.docx]

**
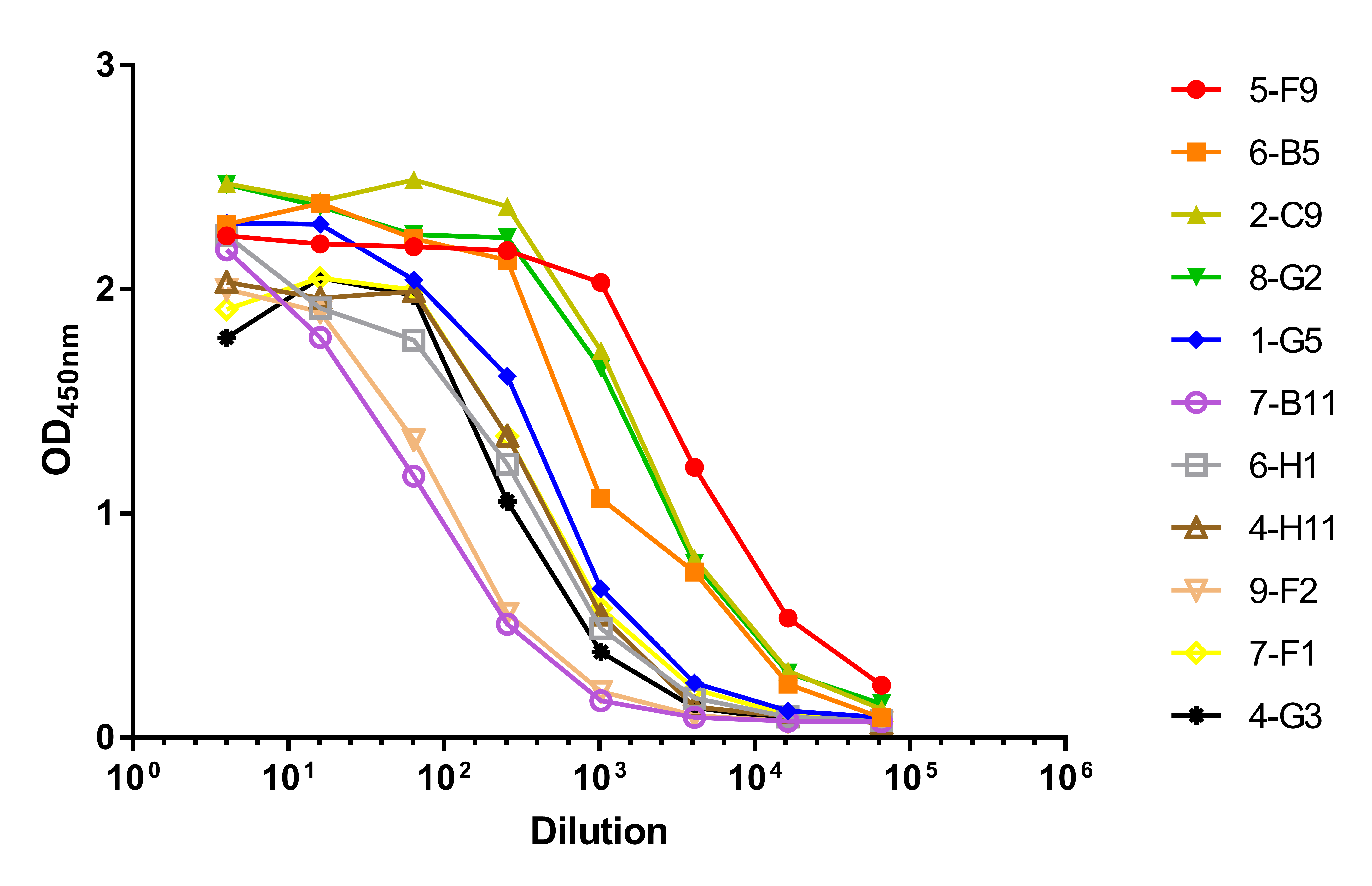
**

**Supplementary Fig1 Dilution assay of hybridoma antibodies against PEDV S1 protein ELISA.** The 11 stable antibody-secreting hybridoma clones were obtained by five stable generations and measured antibody titers through indirect ELISA using the purified PEDV-S1 protein.
